# Genomic Sequencing Surveillance to Identify Respiratory Syncytial Virus Mutations, Arizona, USA 

**DOI:** 10.3201/eid2911.230836

**Published:** 2023-11

**Authors:** LaRinda A. Holland, Steven C. Holland, Matthew F. Smith, Victoria R. Leonard, Vel Murugan, Lora Nordstrom, Mary Mulrow, Raquel Salgado, Michael White, Efrem S. Lim

**Affiliations:** Arizona State University, Tempe, Arizona, USA (L.A. Holland, S.C. Holland, M.F. Smith, V.R. Leonard, V. Murugan, E.S. Lim);; Valleywise Health, Phoenix, Arizona, USA (L. Nordstrom, M. Mulrow, R. Salgado, M. White)

**Keywords:** respiratory syncytial virus, viruses, respiratory infections, RSV, genomic surveillance, antigenic sites, Arizona, United States

## Abstract

We conducted surveillance of respiratory syncytial virus (RSV) genomic sequences for 100 RSV-A and 27 RSV-B specimens collected during November 2022–April 2023 in Arizona, USA. We identified mutations within prefusion F-protein antigenic sites in both subtypes. Continued genomic surveillance will be critical to ensure RSV vaccine effectiveness.

Respiratory syncytial virus (RSV), an RNA virus of the family Pneumoviridae, causes acute respiratory infections, primarily in children, adults with severe lung disease, and elderly persons (*1*). The United States experienced an early surge in RSV cases during the 2022–2023 respiratory pathogen season, coinciding with high levels of influenza and SARS-CoV-2 infections (*2*). In Arizona, USA, numbers of laboratory-confirmed RSV cases increased beginning in September 2022, peaked in mid-November 2022, and then declined to average levels by March 2023 (Figure, panel A). 

**Figure:**
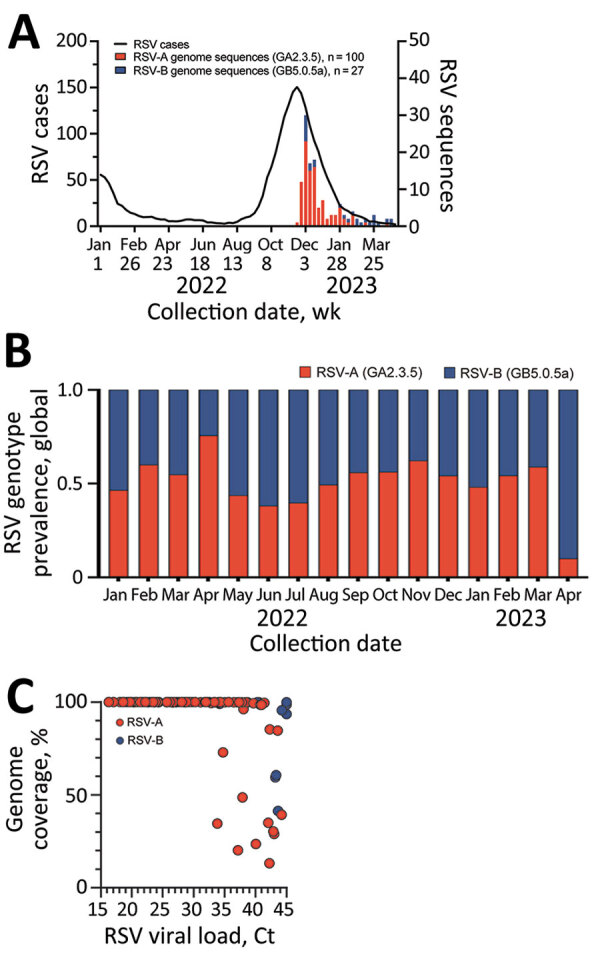
Genomic sequencing analysis of RSV in Arizona, USA, 2022–2023. A) Five-week moving average of PCR-confirmed RSV detections in Arizona reported to the National Respiratory and Enteric Virus Surveillance System and RSV sequence counts by genotype obtained for specimens used in this study. B) Relative abundance of RSV-A and RSV-B genotypes shown for all RSV genomes (RSV-A, n = 1,047; RSV-B, n = 941) deposited in GISAID (https://www.gisaid.org) with collection dates January 1, 2022–May 1, 2023, including genotypes obtained for specimens used in this study (RSV-A n = 100; RSV-B, n = 27). C) Reverse transcription PCR Ct values and genome coverage for RSV-A and RSV-B samples. Ct, cycle threshold; RSV, respiratory syncytial virus.

The 2 major RSV subtypes, RSV-A and RSV-B, have distinct antigenic characteristics in the P, N, F, and G proteins (*1*). Each subtype is classified into genotypes based on sequence variability in the G protein (*3*). According to sequencing data from the GISAID database (*4*), global distribution of RSV genotypes during 2022–2023 was split between the GA2.3.5 genotype of RSV-A and the GB5.0.5a genotype of RSV-B (Figure, panel B). However, RSV vaccines are based on the prefusion conformation of the F protein. Because surveillance efforts should focus on whole-genome sequencing to better understand the evolution of the virus and its potential effect on vaccine efficacy, we conducted surveillance of genomic sequences from RSV-A and -B subtypes to identify genetic mutations. This study was approved by the Arizona State University Institutional Review Board (STUDY00011967). 

We tested 127 RSV-positive nasopharyngeal swabs from previous standard-of-care respiratory pathogen testing at Valleywise Health Medical Center, which serves Maricopa County, Arizona, USA. Patients were a median of 22 years of age (interquartile range 2–44 years). We performed genomic surveillance to determine RSV strains circulating in Arizona during the 2022–2023 season. We performed 2 × 150-bp paired-end next-generation sequencing using a hybrid capture enrichment panel (Illumina Respiratory Virus Oligo Panel version 2, https://www.illumina.com). We used Trim Galore version 0.6.10 (https://github.com/FelixKrueger/TrimGalore) to quality filter and adaptor trim sequencing reads, mapped the reads to RSV-A and RSV-B reference sequences (GISAID accession nos. EPI_ISL_412866 and EPI_ISL_165399) using Burrows-Wheeler Aligner version 0.7.17-r1188 (https://bio-bwa.sourceforge.net), and generated consensus sequences using SAMtools version 1.17 (https://github.com/samtools/samtools/releases). We assembled 92 RSV-A (GA2.3.5 genotype) and 24 RSV-B (GB5.0.5a genotype) complete genome sequences and 8 RSV-A (GA2.3.5) and 3 RSV-B (GB5.0.5a) partial genome sequences (GenBank accession nos. OR143134–250; GISAID accession nos. EPI_ISL_17808760–814) (Figure, panel A). 

To determine RSV viral load, we performed quantitative reverse transcription PCR using pan-HRSV assays that recognize both subtypes RSV-A and RSV-B (*5*). The mean RSV cycle threshold (Ct) value was 29.83 (SD 7.44). We found that specimens with viral load Ct ≤33 yielded 99%–100% genome coverage (Figure, panel C). Whole-genome phylogenetic analysis (Nextclade version 2.14.1, https://clades.nextstrain.org) showed that RSV-A sequences from Arizona clustered in clade GA2.3.5, indicating >3 independent introductions of RSV-A into Arizona (Appendix Figure 1). RSV-B sequences from Arizona formed a monophyletic group in clade GB5.0.5a, indicating a single introduction of strains locally transmitted within the state (Appendix Figure 1). Our findings were consistent with RSV investigations in Massachusetts (*6*) and Washington (*7*), USA, both of which suggested the atypical increase in cases during the 2022–2023 season resulted from multiple introductions of extant lineages, not a divergent RSV lineage with increased virulence or transmissibility. 

The US Food and Drug Administration has approved 2 RSV vaccines for persons >60 years of age, both based on the RSV prefusion F protein. Arexvy (GlaxoSmithKline, https://www.gsk.com) is monovalent and Abrysvo (Pfizer, https://www.pfizer.com), bivalent (*8*,*9*). Most host antibodies target 6 antigenic sites (Ø–V) of the F protein (*10*). Within the F-gene sequences from RSV genomes from Arizona, we identified 7 nonsynonymous substitutions in antigenic sites I, II, IV, and V of RSV-A and 5 nonsynonymous substitutions in antigenic sites Ø, I, II, and V of RSV-B. We found each RSV-A genome mutation in <10% of samples. Most RSV-B genome mutations were more frequent; we found only 1 rare single-nucleotide polymorphism. Mutation frequencies were comparable with trends in recent global RSV genome sequences (Table). RSV-B S190N, S211N, and S389P mutations specifically have become increasingly dominant since 2020. Mutability at residue 389 was shared between subtypes, and each subtype had a preferential amino acid. Finally, we located the mutated residues on the prefusion F-protein crystal structure (protein data bank 7KQD). Many mutated residues were exposed on the F protein surface, suggesting they might interfere with antibody recognition (Appendix Figure 2). 

**Table:** Nonsynonymous amino acid substitutions in RSV-A and RSV-B F protein antigenic sites found in genome sequences in Arizona, USA, during 2022–2023 compared with GISAID global genome sequences

Mutation	Antigenic site	Global frequency, no. (%)				Arizona frequency,† no. (%)
		2020	2021	2022	2023†	
RSV-A		n = 967	n = 1,144	n = 740	n = 115	n = 92
I57V	V	0	0	19 (3)	1 (1)	2 (2)
I59V	V	2 (<1)	1 (<1)	0	0	1 (1)
S276N	II	29 (3)	51 (4)	142 (19)	14 (12)	4 (4)
V379A	I	0	0	1 (<1)	0	8 (9)
L381I	I	0	0	0	0	1 (1)
P389S	I	0	5 (<1)	3 (<1)	0	2 (2)
K470R	IV	0	0	0	0	1 (1)
RSV-B		n = 305	n = 812	n = 720	n = 126	n = 24
R42K	I	1 (<1)	15 (2)	61 (8)	16 (13)	18 (75)
S190N	V	9 (3)	191 (24)	400 (56)	116 (92)	24 (100)
S211N	Ø	1 (<1)	195 (24)	399 (55)	116 (92)	24 (100)
E378D	III	1 (<1)	0	0	0	2 (8)
S389P	I	0	193 (24)	412 (57)	116 (92)	24 (100)
*GISAID, https://www.gisaid.org; RSV, respiratory syncytial virus. †Through April 30, 2023.						

Although RSV remains a substantial clinical burden, approved RSV vaccines reduce the risk of lower respiratory tract illness. By tracking RSV evolution, we can improve design of vaccine formulations to improve effectiveness. Our study was limited because we lacked understanding of potential functional consequences from mutations in F-protein antigenic sites. Although the G protein is under greater selective pressure and has higher mutation rates (*3*), observing its evolutionary trajectory in context with the F protein will be critical. Our study demonstrates the value of using whole-genome sequencing to identify genetic mutations in respiratory pathogens, including RSV, to ensure ongoing effectiveness of RSV vaccines. 

Appendix Additional information about a study of respiratory syncytial virus in Arizona, USA, 2022–2023
