# Supplementary material for: Genomic Sequencing Surveillance to Identify Respiratory Syncytial Virus Mutations, Arizona, USA
Source: Emerg Infect Dis. 2023 Nov;29(11):2380–2. doi: 10.3201/eid2911.230836 (PMC10617361; doi:10.3201/eid2911.230836)
Supplement: Appendix — Additional information about a study of respiratory syncytial virus in Arizona, USA, 2022–2023 [file 23-0836-Techapp-s1.pdf]

Article DOI: https://doi.org/10.3201/eid2911.230836

EID cannot ensure accessibility for supplementary materials supplied by authors. Readers who have difficulty accessing supplementary content should contact the authors for assistance.

Genomic Sequencing Surveillance to Identify Respiratory Syncytial Virus Mutations, Arizona, USA

Appendix

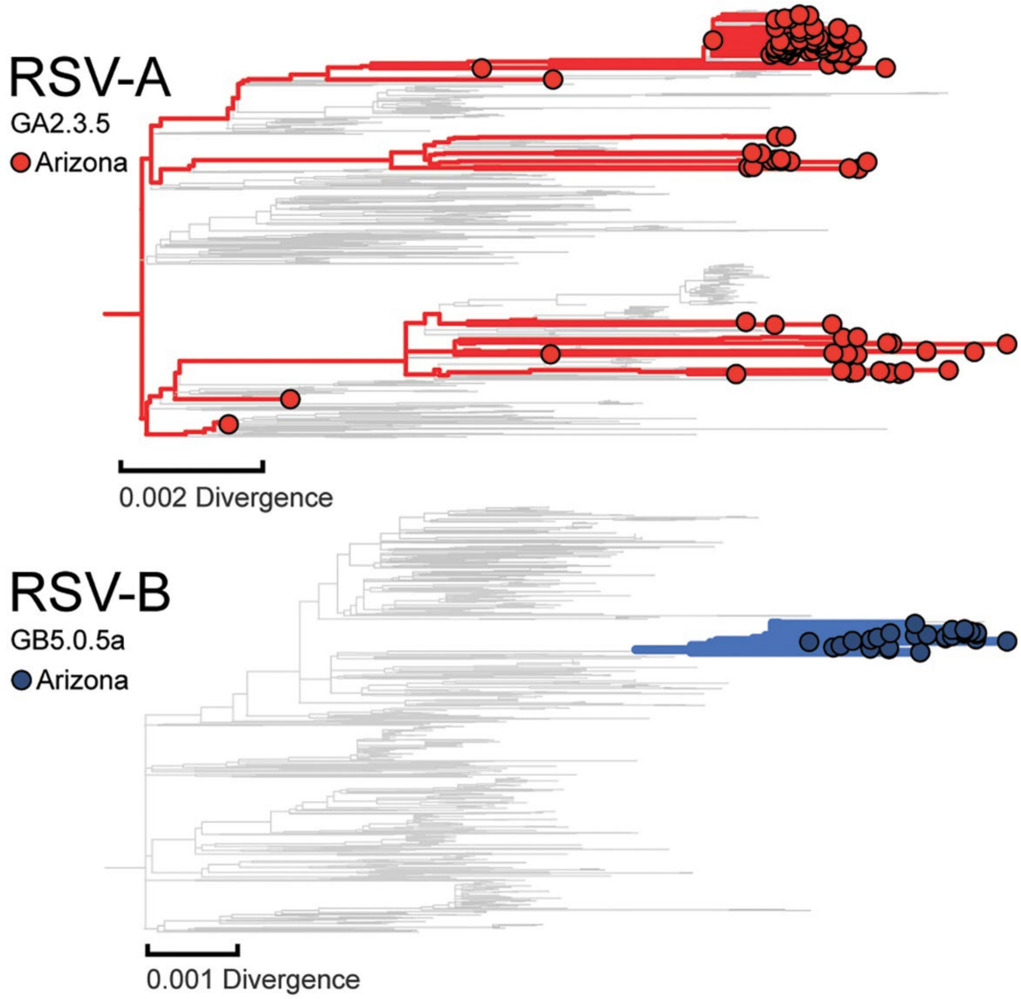

Appendix Figure 1. Phylogeny of RSV-A GA2.3.5 (top) and RSV-B GB5.0.5a clades (bottom) of respiratory syncytial virus (RSV) in Arizona, USA, 2022–2023. Nodes and colored branches highlight sequences from Arizona.

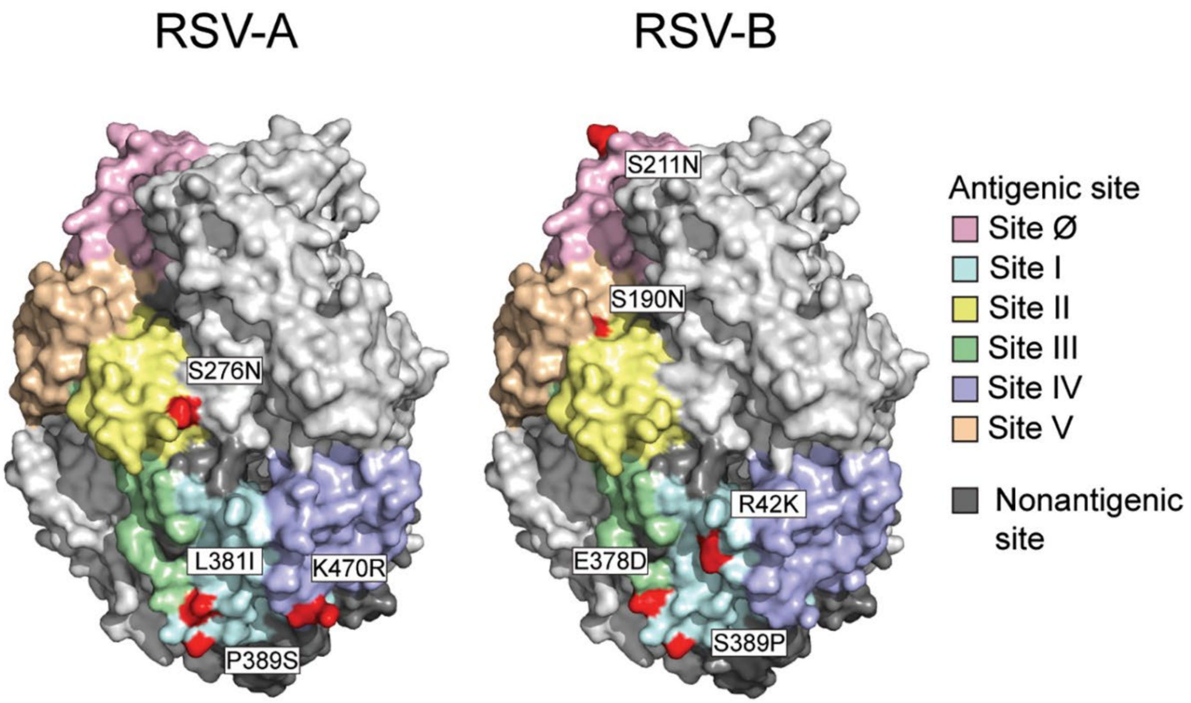

Appendix Figure 2. Structure of RSV prefusion F protein with surface exposed RSV-A (left) and RSV-B (right) single nucleotide polymorphisms found in Arizona sequences labeled and highlighted in bold red. One protomer of each trimer model is colored by antigenic site, 2 additional protomers shown in white.
